# Using Wastewater Surveillance Data to Support the COVID-19 Response — United States, 2020–2021

**DOI:** 10.15585/mmwr.mm7036a2

**Published:** 2021-09-10

**Authors:** Amy E. Kirby, Maroya Spalding Walters, Wiley C. Jennings, Rebecca Fugitt, Nathan LaCross, Mia Mattioli, Zachary A. Marsh, Virginia A. Roberts, Jeffrey W. Mercante, Jonathan Yoder, Vincent R. Hill

**Affiliations:** ^1^Division of Foodborne, Waterborne, and Environmental Diseases, National Center for Emerging and Zoonotic Infectious Diseases, CDC; ^2^Division of Healthcare Quality Promotion, National Center for Emerging and Zoonotic Infectious Diseases, CDC; ^3^Ohio Department of Health; ^4^Utah Department of Health.

Wastewater surveillance, the measurement of pathogen levels in wastewater, is used to evaluate community-level infection trends, augment traditional surveillance that leverages clinical tests and services (e.g., case reporting), and monitor public health interventions (*1*). Approximately 40% of persons infected with SARS-CoV-2, the virus that causes COVID-19, shed virus RNA in their stool (*2*); therefore, community-level trends in SARS-CoV-2 infections, both symptomatic and asymptomatic (*2**)* can be tracked through wastewater testing (*3*–*6*). CDC launched the National Wastewater Surveillance System (NWSS) in September 2020 to coordinate wastewater surveillance programs implemented by state, tribal, local, and territorial health departments to support the COVID-19 pandemic response. In the United States, wastewater surveillance was not previously implemented at the national level. As of August 2021, NWSS includes 37 states, four cities, and two territories. This report summarizes NWSS activities and describes innovative applications of wastewater surveillance data by two states, which have included generating alerts to local jurisdictions, allocating mobile testing resources, evaluating irregularities in traditional surveillance, refining health messaging, and forecasting clinical resource needs. NWSS complements traditional surveillance and enables health departments to intervene earlier with focused support in communities experiencing increasing concentrations of SARS-CoV-2 in wastewater. The ability to conduct wastewater surveillance is not affected by access to health care or the clinical testing capacity in the community. Robust, sustainable implementation of wastewater surveillance requires public health capacity for wastewater testing, analysis, and interpretation. Partnerships between wastewater utilities and public health departments are needed to leverage wastewater surveillance data for the COVID-19 response for rapid assessment of emerging threats and preparedness for future pandemics.

In nearly 80% of U.S. households, fecal waste is transported from homes to wastewater treatment plants within hours (*7*). Wastewater represents a pooled community stool sample that can provide information on infection trends in the community served by the sewer network (sewershed), which can range in size from fewer than 2,000 to >3 million persons. Wastewater surveillance data provide information about symptomatic and asymptomatic SARS-CoV-2 infections. The accuracy of this surveillance approach is not influenced by health care access or clinical testing capacity. In addition, SARS-CoV-2 infection trends in a community can be detected in wastewater before other COVID-19 surveillance metrics, such as case reports and hospital admissions (*3*–*6*). To build sustainable national wastewater surveillance capacity, CDC focused NWSS development in four areas: 1) offering technical assistance to implementing jurisdictions; 2) creating a data portal for centralized data submission and standardized data analysis and visualization; 3) coordinating communities of practice* to share best practices among health departments, public health laboratories, and utilities; and 4) building epidemiology and laboratory capacity for wastewater surveillance at health departments. 

In the NWSS framework, health departments coordinate sample collection and laboratory testing, upload data to a CDC platform for analysis, and use findings to guide public health actions. To facilitate robust analysis, data comparability, and appropriate interpretation, the NWSS data platform receives SARS-CoV-2 RNA measurements and quality control data, performs automated data quality checks, adjusts SARS-CoV-2 concentrations for wastewater composition and method performance,^†^ and performs regression analyses from serial measurements to classify SARS-CoV-2 wastewater trends. A dashboard available to public health departments provides data visualization (Supplementary Figure, https://stacks.cdc.gov/view/cdc/109216). NWSS activities were reviewed by CDC and were conducted consistent with applicable federal law and CDC policy.^§^

Initial SARS-CoV-2 wastewater surveillance efforts in the United States were led by academic researchers, commercial laboratories, and wastewater utilities, with limited public health engagement. Laboratories used diverse testing methods with different performance characteristics, which complicated data analysis and interpretation for public health action. SARS-CoV-2 wastewater sampling strategies need to balance public health objectives with available resources. The objective is to detect the presence of SARS-CoV-2 within a population or to monitor infection trends using changes in the concentration of SARS-CoV-2 RNA in wastewater. Sewersheds can be selected to achieve coverage for a specific proportion of the population, to provide data on communities at higher risk for COVID-19, or to provide data where clinical testing is limited. Laboratories prepare samples, concentrate and extract RNA, and measure SARS-CoV-2 RNA concentrations using methods optimized for wastewater (*8*), emphasizing the need to build environmental microbiology capacity and expertise at public health laboratories (*9*). Data need to be available within 5–7 days of sample collection to ensure timely application for response decisions. The following descriptions of states using wastewater surveillance data to track community trends of SARS-CoV-2 infection provide examples of public health action taken in Ohio and Utah. 

The Ohio Wastewater Monitoring Network, a statewide network launched in June 2020, was designed to provide early warning of increasing SARS-CoV-2 infection in communities and continues to support and guide local and state public health actions to mitigate COVID-19. The network, managed by the Ohio Department of Health (ODH), is a collaboration between local, state, and federal agencies and academic institutions. Twice weekly, 24-hour composite wastewater samples are collected at the influents of 65 wastewater treatment plants, which serve nearly half the state’s population and represent communities ranging in size from 3,300 to 655,000 persons. Samples are tested by six participating universities and one commercial laboratory. The wastewater is analyzed within 3–4 days of collection, and results are uploaded daily for display on the state’s web-based coronavirus dashboard.^¶^

Before joining NWSS, ODH established criteria for notifying local health districts (LHDs), utilities, and community leaders of substantial increases of SARS-CoV-2 levels in wastewater (reported as gene copies per day). A notification is emailed if ODH observes a tenfold increase in SARS-CoV-2 levels over those detected in the past two samples. These local groups use this early warning information, alongside wastewater dashboard data, to guide actions to limit further disease spread. Wastewater increase notifications are also shared with state testing and contact tracing teams that offer assistance to LHDs; since June 2020, approximately 500 notifications have been generated. A toolkit** of social media and press resources and answers to frequently asked questions is provided to LHDs to assist in communicating information to the public.

Utah began piloting SARS-CoV-2 wastewater surveillance in March 2020 as a collaboration between the Utah Department of Environmental Quality, the Utah Department of Health (UDOH), and several academic laboratories. Sampling was extended to wastewater facilities across the state in July 2020. Samples are currently collected twice a week from 42 facilities that serve approximately 80% of the state’s population. Utah developed a public dashboard^††^ and currently disseminates a summary of new data at least twice weekly to local health departments, UDOH leadership, and other pandemic response personnel. Utah’s wastewater surveillance data have been used to help direct clinical testing resources to areas experiencing increased SARS-CoV-2 RNA levels in wastewater. Beginning in 2021, wastewater data are one of the main components of a ranking system to determine where to send mobile testing teams. Wastewater data have also been used when interpreting clinical case surveillance data. As an example, declining clinical case rates occurred in some regions in July 2020. However, the number of persons being tested was also decreasing in some of these areas, raising the possibility that the case rate decreases were a result of declining clinical testing volume. Consistently decreasing SARS-CoV-2 RNA levels in wastewater indicated that the declining case rates were accurate.

## Discussion

Wastewater surveillance is a valuable tool to guide health departments’ COVID-19 response efforts. State health departments have used wastewater data to allocate testing resources, evaluate possible irregularities in traditional surveillance, refine health messaging, and forecast clinical resource needs at the community level. CDC developed NWSS to coordinate and build capacity for wastewater surveillance and transform independent local implementation efforts into a robust, sustainable national surveillance system.

The findings in this report are subject to at least four limitations. First, wastewater surveillance cannot provide data in communities and facilities that are not served by municipal sewer systems. Second, interpretation of the results is limited in communities with highly transient populations, such as industrial or tourist regions. Third, the limit of detection for wastewater surveillance (e.g., the fewest infections in a community that can be reliably detected in wastewater) is not well established. Thus, wastewater surveillance cannot be used to determine whether a community is free from SARS-CoV-2 infections. Finally, wastewater system design and operations, such as pretreatment of incoming wastewater, can affect test results. Communication with wastewater utility partners is important in identifying system characteristics that might affect testing.

NWSS participation is expected to grow as health departments and public health laboratories develop wastewater surveillance coordination, epidemiology, and laboratory capacity. As of August 2021, 43 public health departments are using CDC funds to support wastewater surveillance activities, 32 state and local health departments are participating in the public health community of practice and nine states are reporting data to NWSS. NWSS provides a robust, highly adaptable platform for community-level disease surveillance that can be expanded to collect data on multiple pathogens, such as antibiotic resistant bacteria and enteric pathogens, and leveraged for rapid assessment of emerging threats and preparedness for pandemics.

**Summary**

**What is already known about this topic?**

Wastewater surveillance measures community infection trends. The accuracy of this surveillance approach is independent of health care–seeking behavior, health care access, or testing capacity. The National Wastewater Surveillance System (NWSS) is a 43-jurisdiction, CDC-coordinated system for SARS-CoV-2 wastewater surveillance.

**What is added by this report?**

Wastewater surveillance data have been used to deploy clinical testing resources, investigate possible irregularities in traditional surveillance, refine health messaging, and forecast clinical resource needs.

**What are the implications for public health practice?**

NWSS provides community-level surveillance data that complement traditional surveillance and facilitate earlier, focused health department intervention and support in communities experiencing increasing trends in wastewater SARS-CoV-2 concentrations. Community-level wastewater surveillance data can be leveraged for rapid assessment of emerging threats and preparedness for future pandemics.

## Footnotes

*
https://www.cdc.gov/phcommunities/index.html

†
https://www.cdc.gov/healthywater/surveillance/wastewater-surveillance/data-reporting-analytics.html#data-analytics

§ 45 C.F.R. part 46.102(l)(2), 21 C.F.R. part 56; 42 U.S.C. Sect. 241(d); 5 U.S.C. Sect. 552a; 44 U.S.C. Sect. 3501 et seq.

¶
https://coronavirus.ohio.gov/wps/portal/gov/covid-19/dashboards/other-resources/wastewater

**
https://coronavirus.ohio.gov/wps/portal/gov/covid-19/healthcare-providers-and-local-health-districts/for-local-health-districts-and-governments

††
https://deq.utah.gov/water-quality/sars-cov-2-sewage-monitoring
